# Depressive disorders in Thai medical students: an exploratory study of institutional, cultural, and individual factors

**DOI:** 10.5116/ijme.5fbe.4ce5

**Published:** 2020-12-26

**Authors:** Winitra Kaewpila, Papan Thaipisuttikul, Tantawan Awirutworakul, Karn Jumroonrojana, Umporn Pitidhammabhorn, Fred Stevens

**Affiliations:** 1 Chakri Naruebodindra Medical Institute, Faculty of Medicine, Ramathibodi Hospital, Mahidol University, Samut Prakan, Thailand; 2 Department of Psychiatry, Faculty of Medicine, Ramathibodi Hospital, Mahidol University, Bangkok, Thailand; 3 Department of Psychiatry, Faculty of Medicine, Ramathibodi Hospital, Mahidol University; 4 Department of Educational Development and Research, Faculty of Health, Medicine and Life Sciences, Maastricht University, The Netherlands

**Keywords:** Medical students, depression, medical student wellness, academic achievement, learning environment

## Abstract

**Objectives:**

This exploratory qualitative study conducted among Thai medical students aimed to investigate factors related to the development of medical students' depression and how these factors interact in contributing to depressive disorders.

**Methods:**

Forty-three undergraduate medical students of the six-year Doctor of Medicine program were identified as having moderate to severe depression on an annual depression screening. From these, eighteen students agreed to participate in individual in-depth interviews. Transcriptions of the interviews were analyzed by independent reviewers using a thematic analysis approach.

**Results:**

Among 43 participants screened as having moderate-to-severe depression, major depressive disorder and adjustment disorder were 9.3% and 14.0%, respectively. Reported factors related to medical students' disorders were personal vulnerabilities, medical educational administration, academic achievement, learning environment, intrinsic motivation, self-care and self-management, relationship, and community. In particular, lack of social support and relationship problems were mentioned among those with more severe and persistent symptoms. Protective factors were social support, positive relationships, a growth mindset, spiritual and mindfulness practices, and an adequate mentoring program.

**Conclusions:**

Medical students' depression and suicidal ideations are significant concerns in Thai medical education. Besides personal vulnerabilities, high expectations, the value placed on academic achievement, and relationship problems can precipitate the onset of depressive disorders, if not being properly addressed. The 4P framework of predisposing, precipitating, perpetuating, and protective factors are suggested to understand the onset and development of students' depressive disorders and to identify targets for institutional and educational intervention.

## Introduction

Depression among medical students is becoming a major concern in medical education. It manifests itself in changes in emotions, behaviors, eating and sleeping patterns. Depression negatively affects concentration, memory, and the ability to study and socialize with others, being at risk for suicide ideation.^1^^-^^4^ Depression may also affect medical students' ability to understand and empathize with patients.^5^ According to one meta-analysis, a worldwide prevalence of depression among medical students has been estimated to be around 28%.^6^ This number was the same as the 28% prevalence of depression among Thai medical students.^7^ It is higher compared to age-matched peers in the general population and continues to increase.^8^^,^^9^ High numbers are not uncommon in Asia. The mean prevalence of depression amongst medical students in China is 32.74%. Associated factors are female gender, year of medical study, grade level, satisfaction in current major, residence, and monthly income.^10^

Studies are done primarily used quantitative methodologies.^11^^,^^12^ Only a few earlier qualitative studies explored factors associated with medical students' stress, anxiety and depression.^13^ These include stressors at the individual level such as excessive workload, difficulties with studying and time management, conflicts in work-life balance and relationships, medical school peer relations, health concerns, and financial stressors.^13^^,^^14^ Stressors measured at the institutional level include medical school administrative failures, concerns about lack of assistance with career planning, and assessment-related performance pressure.^13^^,^^14^

Although recently more studies have been conducted using qualitative approaches, the majority is based on depression screening tools.^13^ No qualitative studies are known for having been designed with medical students confirmed DSM-5 diagnosis as a basis. The advantage of such an approach is that it will provide in-depth understanding of factors and their mechanisms contributing to mental disorders as identified and verified by psychiatrists. Further development of interventions and prevention will benefit from it. This study aims to investigate a) factors related to medical students' depression, b) how these factors interact in contributing to depression as diagnosed by psychiatrists, according to DSM-5 criteria.

## Methods

### Study design

This is an exploratory qualitative study with thematic analysis.^15^ Qualitative methodology was used to reach a thorough understanding of medical students' experiences and perceptions. To determine the prevalence of depressive disorders and to explore which factors related to student's depression, eighteen individual in-depth interviews were conducted with medical students scoring high on depression screening.

### Participants and settings

The study took place at the Faculty of Medicine Ramathibodi Hospital, a University hospital in Mahidol University, Bangkok, Thailand. Eighteen undergraduate medical students of the six-year Doctor of Medicine program were invited to participate. This was done in two steps. First, all medical students from year 2-6 completed the annual screening protocol for depression by the Department of Medical Education, using the PHQ-9 depression scale.^16^ First-year medical students studying outside Ramathibodi hospital were excluded. From 451 medical students who took the survey screening, 43 were identified as having a moderate to severe depression (scored 15 or higher on PHQ-9).^16^ From these 43 students, the researchers contacted 35 students, who agreed in the survey to allow a call from researchers either to receive a psychological intervention or to participate in this research. Eighteen medical students agreed to participate in individual interviews. The reasons students declined to participate were that they already were in treatment and/or their symptoms subsided at the time of interview. All participants were provided information about the research project, the purpose of the interview, and ensured anonymity and confidentiality. Subsequently, students who agreed to participate gave consent and entered the interview process without any compensation. The study was approved by the Ethics Committee of the Faculty of Medicine, Ramathibodi Hospital, Mahidol University.

### Data collection methods

Individual Interviews were conducted by four psychiatrists, PT, WK, TA and KJ, during January-March 2018. The first part contained questions regarding background characteristics and psychiatric assessment to arrive at any DSM-5 diagnosis. The second part consisted of semi-structured interviews to explore factors associated with depression, both in terms of personal and psychosocial factors, their stress, concerns, motivation and views on the meaning and values of life and becoming a medical professional. Each interview lasted 45-60 minutes and was recorded and transcribed verbatim. Identifiable labels in the recording were removed before data analysis and eventually were deleted after the analysis was completed. After the interview, psychiatrists assessed whether students needed further psychiatric management. If additional management was necessary, the team transferred care to another psychiatrist or to the Department of Student Affairs.

### Analysis

The transcribed interviews were analyzed using a thematic analysis approach.^15^ In the first step, the data were read several times, guided by the research questions. Initial codes were generated and collected. Next, the researchers searched for patterns and themes emerging from codes and data. Then the themes were reviewed by independent reviewers. The defining and labeling of themes were discussed among the authors to reach an agreement. In the second step of the analysis, all data and initial themes were revisited to look for more underpinning causes to come to final conclusions.

### Reflexivity

Five researchers (WK, PT, TA, KJ, UP) are all psychiatrists. All of them were not acquainted with any of the participants. In terms of researchers' personal background, all were once medical students, which may influence perceptions as insiders, but also can give unique insights into the essence of the phenomenon under study.^17^ Being a medical educator is also a social position.^17^^,^^18^ One needs to be aware of not to focus or filter some aspects of insights from participants. In addition, being medical educators conducting the study with medical students, especially in the Asian culture, there are also issues of power and rank differences that may influence the participants' responses in the process. We did make sure to clarify our objectives and agenda with the study and made sure students were aware of confidentiality, safety and rights to participate in or withdraw from the study at any time. A supervising researcher (FS) is an educationalist and medical sociologist, among others, specialized in research methodology and qualitative methods. He also graduated with a Master of Health Professions Education. He had an outsider perspective, not being involved with the faculty of Medicine Ramathibodi Hospital. This insider/outsider status of the researchers allowed us to understand and interpret the findings from different perspectives.^17^ All authors declare no conflict of interest in this study.

## Results

After having completed interviews and psychiatric evaluation in 18 participants, according to DSM-5 criteria, four were diagnosed with major depressive disorders (MDD) and six were diagnosed with adjustment disorders (AjD). At the time of the interviews, of the four medical students with MDD, two had remissions. The other two had partial remission to depression. From 43 participants who were screened as moderate-to-severe depression by PHQ-9, the percentage of MDD and adjustment disorder were 9.3% and 14.0%, respectively. Regarding the factors related to medical students' depression, we identified seven themes that contributed to medical students' depression, as described below.

### Personal vulnerabilities

Personal vulnerabilities can be genetic factors such as a family history of mental health difficulties which were founded in two students diagnosed with MDD. One student has a mother with the depressive disorder, while another one mentioned the history of his mother's suicidal attempts. Hardship in life's experiences, such as parental divorces and family conflicts were also reported. Medical students frequently brought up their personality traits as causes for their stress and depression. These traits are perfectionism, anxiousness, unsocialized personalities and sensitivity to rejection. In addition, insufficient coping skills, such as lack of self-management, avoidance and social isolation, were recognized as students' problems. One student described her excessive anxiety with a clinical study,

"...I am always anxious about studying, in surgery rotation. I was really nervous every Sunday evening since I have to move to a new unit. I felt like it's all in my head because on Monday, it's okay, nothing bad happens but I would still get really anxious and restless every week…" (student no.6, female, AjD).

Another student mentioned his avoidance of coping styles that could put him into troubles with studying,

“...when I don't like the subject, I don't want to study, I would read and study only the part I like...when the national license exam approached, I was stressed because I didn't understand what my friends were talking academically. I don't want to fail but I just can't study, so I started playing video games as a reward for studying. But then I found myself just playing games, not studying at all, I felt guilty but I can't stop, then for a while, I felt bored with everything. I didn't play games. I didn't study. I just slept and ate…" (student no.10, male, MDD)

### Medical educational administration

Medical students described their medical education containing an abundant amount of content knowledge to be absorbed in a tight schedule with time constraints and frequent high-stake examinations. As one participant mentioned,

"I felt that learning medicine was so scary. I had exams every week or two, and I did not know when I was going to fail or might get cut off, it was like I could die any minute. I felt on edge all the time." (student no.12, female, AjD)

The curriculum organization might require students who fail one course to wait a year to repeat the same course. These difficulties add high levels of stress to vulnerable students and make it harder for them to recover. As one student described that his failing in one course leads to repeating a year, causing depressive episodes:

“...I failed and had to repeat the third year again. I was so weak then… I was so sad and couldn't do anything. I ate a lot and gained almost 15 kilos, that period last six-seven month. I realized that I was having MDD that time while I later studied psychiatric rotation... I'm not scared much of the content or the exam, but the main worry is that once I fail, the system wouldn't help, it never helps or allows the student (who failed) to recover..." (student no.4, male, MDD)

On the other hand, having a student's support system can help students adjust to their problems. For example, one student mentioned the importance of the mentoring program and receiving advice from a mentor,

"I really like the mentoring program. I talk to my mentor, and it helps...I really want to tell you that there are students with difficulties that you don't know about. I can't figure out ways to let the teachers know and help those students with problems. But I do know there are a lot of them" (student no.15, male, AjD)

### Academic achievement

Academic under-performance, such as failing a course, might trigger mental distress, as mentioned above. Medical students are extremely concerned about their grades. They strive to achieve their best academic outcome to guarantee opportunities and professional achievements in the future. As one student mentioned:

"Grades are always highly important for me. After I graduate, there's only one thing left, my grade report. No one will care how much effort I have put in if the grade turns out bad." (student no.11, female, AjD)

Academic achievement in the pre-clinical years may be expressed in terms of grades or assessment reports. Meanwhile, in the clinical years and in addition to grades, there are also workplace-based performance assessments or feedback and comments from teachers or residents during their clinical practice such as during ward rounds, bedside teaching, etc. These performance assessments put a lot of pressure on students and directly affect students' perception of their academic achievement. Constantly seeking for approval, self-assessment and being self-perceived as incompetent are the most common concerns for clinical year students. As one student reported:

“There were good days and bad days…I would always observe others' reactions during ward rounds. There were so many people, friends, seniors, externs, residents, and attending. On the day that I did well, and everyone was happy with my presentation, I would feel happy too. But, if one day I perform worse than I usually did and the attending was not happy with my presentation, all my friends would get negative feedback as well. That negative feedback might spread to residents too, and they were not happy. I really felt that I was a failure!" (student no.11, female, AjD)

### Learning environment

The learning climate and learning environment are highly competitive. Students felt stressed and pressured by fellow students' performance. As one student mentioned,

"It is competitive. Each student studies by his own, the grading is norm-referenced. So we have to kill each other down, I only got B, B+, C, C+. Overall, they don't seem to support each other. That's how our society is like, everything has changed…" (student no.10, male, MDD)

In addition, some of the students have adjustment problems with the new environment in their clinical years, especially the 4th year, which is the first clinical year. One female student described her problem adjusting to new people in the clinical workplace and feelings of a depleted sense of belonging because she couldn't socially and informally fit in with the ward-round group.

"I felt down in 4th year because we had to be in a small group with new friends, new residents, new teachers. We have to get along with them. There are many moments that I felt out of place and isolated. They did not do something bad, they just hung out, joked around or talked about academics. I can't catch it and seem a bit odd. I try to observe and mimic, but it seems unnatural, I try too hard every day, and it's so exhausting, and I feel awful..." (student no.18, female, AjD)

Students also experienced a lack of support from faculty members and felt discomfort with seniority and ranking issues. One student mentioned,

"It depends, some medical teachers don't seem to care about students at all. But even when some do pay attention to us, there still is a gap...like, social status and rank, you know, rank for teachers, residents and for us students. I felt their seniority, and I cannot talk casually." (student no.16, male, AjD)

### Intrinsic motivation

Lack of intrinsic motivation to be a doctor was not uncommon among the participants. A number of students described they never wanted to study medicine in the first place. Since medical students in Thailand usually enter medical school immediately after they graduate from high school, they have no real exposure to medical school or any experience with the profession or hospital. For status reasons, family and supervisors in high school always encourage high-performance students to study medicine. Therefore, lack of intrinsic motivation may result in students not having goals and objectives in learning and missing a self-driven force to overcome obstacles exposed in medical schools. As one participant described it,

"My parents wanted me to go to medical school only! They did not leave any other choice. I'm quite submissive. I don't like to argue with them. At first, I was thinking of something else, but when I told them, they seemed to be very upset. I did not want that situation to happen again...Truthfully, I got into medical school because of their request. I just followed their request." (student no.15, male, AjD)

On the other hand, when students become clear with their goals and future, their sense of direction increases intrinsic motivation and lessens distress. One student was getting better after she confronted her concern of the future, and made a decision for a career path:

“...I used to have no goal for the future.  I can't imagine myself after I graduate (have to work in rural areas to repay the government's scholarship contract). I have to work very far away from home…but now I have no problem with that, I just decided to have my goal set, and I'm fine. I decided to, at least, finish the scholarship contract. Then if I don't have any other choices, I could resign later, but I do want to become a doctor first." (student no.12, female, AjD)

### Self-care and self-management

Medical students usually spend at least 8 hours per day, 5 days a week, in preclinical years, and up to over 12 hours per day in clinical years, not including on-duty or rounds over the weekends.

Medical students complained about their work-life balance and lack of time for self-care and management. They perceived that medical school takes all their time, and they described having no time for hobbies, recreational activities and social life. One participant explained that,

"It was like I was paying 24-hour attention to the study. Other activities were gone from my life. I lost some relationships and at one point I was concerned about who will still be there in my life." (student no.11, female, AjD)

Moreover, medical students mentioned the decrease in quality of life in other areas, for example, students have decreased sleep duration while on call, and insufficient physical rest. One participant complained that,

"I got to sleep late last night because I had to write a report. I came into class after only one hour of sleep. If I had got good feedback from my teacher, I still would have been OK, but I did not…and it was repeated over and over like this. I was so exhausted." (student no.11, female, AjD)

Having a spiritual and mindfulness practice can help students to be calmer, more positive and better-coping skills. One student described how religious and meditation practices gave him good insights and helped me to realize his purpose in life, which improved his intrinsic motivation to study

"...I learned of Buddhist contemplation about death. I imagined if I'm dying...I think about Buddha and I wish I could do good deeds before I die. I never know when it will happen, so in the meantime I want to do something good for other people and for society, I want to become a doctor to help other people. I started practicing meditation and I am calmer." (student no.16, male, adjustment reaction)

In addition, another student also mentioned using extracurricular activities to distract himself from rumination on negative thoughts.

### Relationship and community

Relationship issues appeared to be major concerns for medical students. They were among the most common factors, besides academic achievement, reported by depressed students. Various problems were identified related to students' depression, such as breaking up with a girl/boyfriend, conflict within the family or within a group of peers and adjusting to the medical community. Many students described romantic relationship problems at the beginning of their depression. As one student reported

"...I went to see my girlfriend at another university, and I found out that my girlfriend was seeing another man and broke up with me on that day. I was devastated. That university is near the river, I remembered walking along the river and having a suicidal thought of jumping into the river. I didn't do it then, but I have been depressed ever since…" (student no.10, male, MDD)

Some students reported peer relationship problems. They felt they had to be a member of a specific group of friends, mostly a self-organized group formed in the first year of medical school. Since the group was formed at an early phase when they weren't familiar with each other's personalities, members might eventually not get along. Conflicts could arise, forcing some members to leave for a new group. This group arrangement is important because it is also used in some formal classes such as gross anatomy classes. Many troubled students have ongoing relationship problems with peers and also family relationship problems. As one student previously diagnosed with MDD described his ongoing relationship problems with peers and family that lead to suicidal ideation,

"...I had a problem with this one friend, and we didn't talk, one day he brought the issue up in front of the whole group. I felt bad and kept thinking about this so that I can't read for the exam on the next day. This also led to an argument with another friend, and I felt worse. I went home and my mom didn't understand me, she started crying...I left the room and up to the deck of the apartment...I stood close to the edge looking down...it looked vast, there were so many peoples. Would it matter if one person was gone...I imagined myself falling down...and then I felt the guilt, so I didn't do it" (student no.8, male, MDD).

Relationship aspects appeared to be the most prominent factors that separate students with MDD from adjustment disorders and those without disorders. Students with more severe and persistent symptoms often describe their lack of social support and tend to use social isolation to cope with their problems. On the other hand, having social support can be protective factors helping students cope with challenges. As one student with diagnosed completed remission, MDD described how support from a friend and family helped him through hard times, giving him an insight and a growth mindset:

"...when I had to repeat one more year, I thought I would never be happy again. But thanks to my family and friends. A junior fellow student gave me good insight, so we became close and took turns giving each other a counsel. He said it's the first time in this internal medicine rotation so I shouldn't be so stressed if I fail, I just have to try harder one more time...and I thought, It's true! I have tried so hard up to this moment, and if I fail, then it's just another two or three months. So, it's alright." (student no.4, male, MDD)

## Discussion

Factors identified in this study as contributing to depression in medical students can be helpful to understand and develop early detection and intervention. These include institutional structures (the unique features of the medical education structure and administration) and the institutional culture (high expectations and value placed on academic achievement and a competitive learning atmosphere), combined with individual factors and personal vulnerabilities (such as genetic factors, perfectionism personality traits and avoidance coping). These factors interact and are manifested in medical students' difficulties with academic achievement, intrinsic motivation, self-care/management and relationships. The institutional culture, strongly reflecting society's norms, values and expectations, appears to be extremely impactful on medical student's stress and depression levels. Perceptions of prestige in medicine, family's expectations and the pressure and competence-oriented medical school culture are seen elsewhere too.^19^ But in the Asian context, medical students seem to be much more compiled with the family's rules, values and expectations, including those from the institutional community. Problems tend to become amplified in the strict hierarchical Thai society, where seniority issues may prevent students from seeking support from faculty staff, adding to further stressors. Evidently, cultural differences may explain differences in prevalence, severity and factors related to medical students' depression, including their help-seeking behaviors.

Regarding the severity of depression, the factors identified causing depression did not differ between students with MDD, adjustment disorder and non-disorder students. However, students with more severe and persistent symptoms and disorders reported more factors combined, more intensity of these, in particular in the areas of problems with relationships and lack of social support. This is consistent with findings from the earlier studies.^20^^,^^21^

As we also wanted to bring attention to the deeper understanding of psychological needs of medical students, the identified factors related to medical students' depression reflect underlying psychological needs such as a) self-worth or sense of competence (academic achievement), b) sense of value and meaning (intrinsic motivation), c) autonomy and a sense of control (self-care and self-management). In addition, relationship issues with family, peers and being able to adapt to the community reflect: d) a need for acceptance and a sense of belonging. Our findings show that these psychological needs are important and consistent with some of the six areas of work-life (workload, control, reward, community, fairness and values) as predictors of burnout development.^22^ These needs also align with concepts in motivational theories such as self-determination theory (competence, autonomy and relatedness)^23^ and control-value motivational theory.^24^ Thus, the issues of medical student's motivation, burnout and depression are interrelated and should be addressed comprehensively. Because of this interrelatedness, and to find best tools for intervention, it may be helpful to look deeper at medical students' motivation, burnout and depression in the context of psychiatric disorders and the 4P framework.^25^

### Student depression and psychiatric disorders: The 4P framework

Psychiatric diagnoses are considered as disorders, not diseases, which means the causes usually are complex and multifactorial. No two people with depression have the same symptoms or the same causes. In our study, psychiatric disorders among students were indicated by significant distress and impairment in personal functioning. Such features can be persistent, remitting or relapsing, and can occur as single or recurrent episodes. Two disorders were commonly found: adjustment disorders and major depressive disorders (MDD). In order to plan for intervention, an understanding of each individual patient is needed, including all factors that may contribute to the disorder. For this, the 4P framework can be a helpful tool. The 4P framework, as developed for psychiatric case formulation and discussion, explains the onset and development of psychiatric disorders.^25^ It distinguishes four factors contributing to depression, labeled as: Predisposing, Precipitating, Perpetuating and Protective factors.

**Figure 1:**
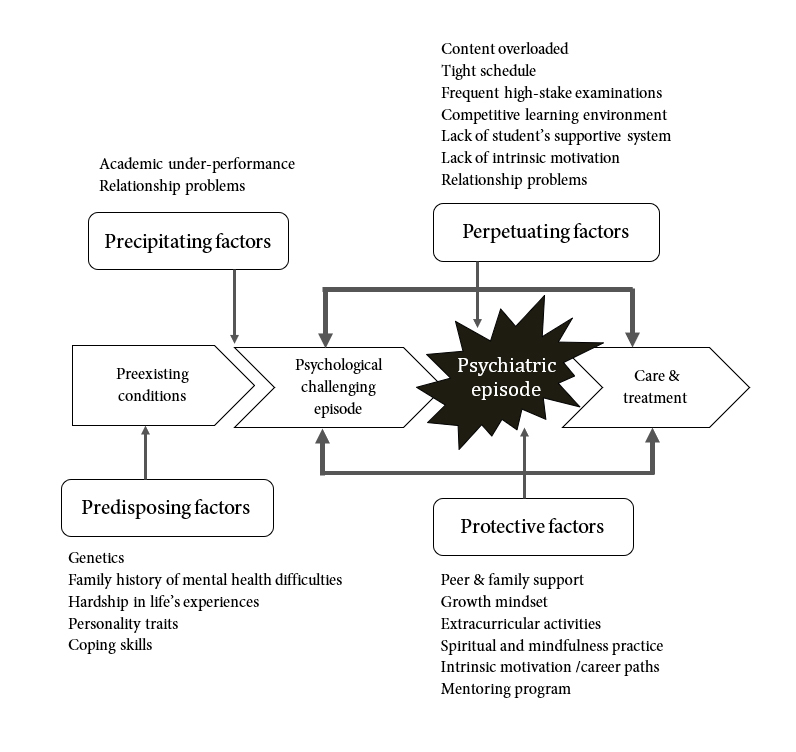
The 4Ps framework illustrates factors related to medical students' depressive disorders

In the context of the 4P framework, the development of medical student's depression can start from the pre-existing individual risks (predisposing factors) combined with external stressors that trigger psychological distress (precipitating factors). How well students can cope with their distresses usually depends on their predisposing risks, other ongoing stressors (perpetuating factors) and protective factors such as relationship and social support. Figure 1 illustrates the 4Ps framework in relation to medical students' depressive disorders, including reported factors.

Predisposing factors are pre-existing risk factors for developing depression. In this study, we could identify personal vulnerabilities as predisposing factors. These are genetic vulnerabilities such as the family history of mental health difficulties, family conflicts and personality traits such as perfectionism, anxiousness, unsocialized personalities and sensitivity to rejection. Also identified insufficient coping skills, such as lack of self-management, avoidance and social isolation in some of our respondents, are part of this category.

Precipitating factors include significant stressors or events that trigger the onset of depression. To this category belong issues related to medical, educational administration, academic achievement, relationship and community, such as academic under-performance, failing courses, being assessed as incompetent, or in problems in relationships with romantic partners, peers or family members.

Perpetuating factors maintain the problem once it has become established. The medical education curriculum and its organization are both creating and maintaining high levels of stress for medical students. More, in particular, perpetuating factors are the overloaded curriculum content, the tight schedule and the frequent high-stake examinations, the curriculum organization which requires students who fail one course to wait a year to repeat the same course including persistent relationship problems. These difficulties add a high level of stress to the vulnerable students and will make it harder for them to recover. Some of the personal vulnerabilities, such as personality traits and insufficient coping skills, can also further perpetuate the problem.

Protective factors (or their absence) can significantly impact on the development of depression and its severity. As our findings have indicated, most important protective factors, which may help medical students to better cope and increase resilience, are social support from friends and family, being able to realize a growth mindset, extracurricular activities, a spiritual and mindfulness practice, realignment of intrinsic motivation and career paths, and mentoring. Students missing these protective factors appeared to have more persistence and severity in their depressive symptoms.

By using the 4P framework for a student's case formulation, medical educators can plan to specifically target each factor involved for intervention and prevention both at the individual and system level. Regarding preventive activities, if factors can be early detected and identified, the risk of adjustment reaction turning to adjustment disorder or MDD may be prevented. Understanding predisposing factors can help medical educators plan for support, for example, setting up a self-development program focusing on facilitating self-reflection, self-awareness, self-care and self-management skills, including spiritual and meditational practices. These will benefit all students. In addition, a mentoring program can support students and provide access to well-being facilities and services. These two interventions are also supported

by the literature.^26^^-^^28^ Understanding precipitating factors is an important part of relapse prevention planning and also provides information for rethinking and redesigning institutional structures and cultures such as the curriculum and schedules. In addition, making students aware of medical school's norms and values will help medical students and other members in the medical community to have realistic expectations.

Understanding perpetuating and protective factors can help prevent or reduce harm from a severe case since once a major disorder is set in motion, it becomes self-perpetuating. It is crucial for medical educators to better understand medical students' needs, risks and the mechanisms that lead to psychological difficulties, in order to plan for individual intervention as well as to optimize instructional and institutional factors. The aims are not only to prevent burnout and depression but also to promote intrinsic motivation, well-being, and to actualize the best learning experiences for all students.

### Limitations of the study

This study has several limitations. Firstly, the actual number of students with disorders might be higher since there were some students who refused to be contacted after the screening, while others, who allowed to be contacted, declined to participate in the study. The reasons students declined to participate were that they already were in treatment and/or their symptoms subsided at the time of interview. Therefore, the actual prevalence of depression in medical students should be the object of further investigation.

Secondly, the small number of interviewed participants might not have allowed us to properly reach theoretical saturation. However, our findings, conducted with participants diagnosed with DSM5 disorders, were consistent with previous studies of stressors among medical students^11^^-^^14^ and also among a group with positive scores on depression screening tools.^29^ Thirdly, this study was conducted in a single institution in Bangkok, Thailand. It means that possible contrasting perspectives from other settings and cultures are absent. A cross-cultural approach could be an interesting topic for further study of factors in the onset and development of depressive disorders among medical students.

## Conclusions

Medical students' depression and suicidal ideations are significant concerns in Thai medical education. Factors related to medical student's depression were interactions among various factors due to individual vulnerabilities and institutional structures and culture. Several strategies can be proposed that may help prevent depression among medical students, such as encouraging self-development programs, improving mentoring programs and a student care system that is easily accessible. Also rethinking medical educational structures and administration to optimize learning experiences tailored to student needs would be appropriate. The 4Ps approach is a promising entrance to better understand individual students and to plan for early prevention and intervention. Medical educators should pay attention to all factors and their functions as predisposing, precipitating, perpetuating and protective factors related to students' stress and depression. Different modalities should be addressed, especially for students with more severe, persistent symptoms and potential risk of suicidal ideations. Future research should be focused at a system-level, exploring institutional factors and the impact of institutional interventions to address medical students' needs, and providing social support. As the institutional culture, reflecting Thai society's norms, values and expectations, appeared to be rather impactful on medical student's stress and depression, it would be interesting to look at these issues from a cross-cultural perspective.

### Acknowledgements

We would like to express gratitude to Asst. Prof. Pongtong Puranitee and Asst. Prof. Sutida Sumrit for their aspiring guidance and contributions on the survey screening process. We also thank Rhianna Goozee, PhD for English editing the first draft of this manuscript and Prof. Dr. Amnuay Thithapandha from Department of Academic Affairs, Faculty of Medicine, Ramathibodi Hospital for English editing the final draft of this manuscript.

### Conflict of Interest

The authors declare that they have no conflict of interest.
